# Effectiveness of Exogenous Ketone Salts in Enhancing Circulating Acetoacetate Levels—A Pilot Study in Healthy Adults

**DOI:** 10.3390/nu17101665

**Published:** 2025-05-14

**Authors:** A. Maleah Holland-Winkler, Andrew R. Moore, Ilya Bederman

**Affiliations:** 1 Department of Kinesiology, Augusta University, Augusta, GA 30909, USA; andmoore@augusta.edu; 2 Department of Genetics and Genome Sciences, Case Western Reserve University, Cleveland, OH 44106, USA; ilya.bederman@case.edu

**Keywords:** ketones, gas chromatography/mass spectrometry, acetoacetate, urinary ketones, beta-hydroxybutyrate

## Abstract

Background/Objectives: Ketone salt (KS) containing a racemic beta-hydroxybutyrate mixture is commonly used as an alternative fuel source as it may lead to improved health and/or performance. We postulate that KS will raise acetoacetate levels and represent the effectiveness of exogenous KS as an energy source. We conducted a pilot study to quantify changes in the circulating acetoacetate following KS and to determine if any changes in acetoacetate were associated with the changes in circulating beta-hydroxybutyrate. Methods: Thirteen adults (21.6 ± 4.3 years old; seven males/six females) completed this randomized, triple-blinded, placebo-controlled, cross-over design study. Participants consumed either KS or flavor-matched placebo with a one-week washout period between supplements. Blood samples were taken before and 30 min after consuming each supplement, and plasma acetoacetate and beta-hydroxybutyrate levels were measured by gas chromatography/mass spectrometry. Results: The consumption of KS resulted in a significant increase in acetoacetate from baseline. The increase in acetoacetate after the KS supplement was significantly greater than that following the consumption of a placebo (↑ 0.57 ± 0.44 mM vs. ↑ 0.07 ± 0.23 mM, *p* = 0.009, d = 0.86), and significantly and strongly related to the change in blood beta-hydroxybutyrate (r = 0.757, *p* < 0.001). Conclusions: Our findings indicate that KS markedly increases plasma ketone body interconversion, presumably to supply peripheral tissues for ATP generation.

## 1. Introduction

Ketones are produced from fat stores to assist in ATP production [1], and thus an alternative fuel source for many organ systems [2]. A substantial rise in ketone body concentrations occurs during advanced starvation or a very-low-carbohydrate ketogenic diet (<50 g of carbohydrate daily) [3]. Some individuals choose to follow a very-low-carbohydrate, ketogenic diet or fast for prolonged periods to establish and maintain a state of nutritional ketosis, which signifies a metabolic shift; the lack of dietary carbohydrate intake reduces insulin secretion, which promotes a breakdown of stored fat and resulting ketone production [4,5]. Fat and ketones then become predominate fuel sources for most organs except for specific areas of the brain and red blood cells which require glucose [6]. Many parts of the brain, however, metabolize ketone bodies which have been shown to supply up to 70% of the brain’s energy requirements [7].

On the other hand, the fed state without carbohydrate restriction leads to a higher insulin/glucagon ratio which prevents ketones from being generated and utilized for energy [5]. To increase circulating ketone levels without restricting carbohydrate in the diet, individuals may opt to supplement with exogenous ketones which are widely available as various mixtures of beta-hydroxybutyrate (βHB) [8,9]. Elevated circulating ketone levels have been shown to positively impact many aspects of health by lowering blood glucose levels, circulating triglyceride levels, blood pressure, and inflammation while improving cognitive function [10,11]. Improvements in these biomarkers may reduce the risk and/or progression of metabolic conditions like type 2 diabetes and cognitive conditions like Alzheimer’s Disease [12,13,14].

Circulating βHB concentration is used as a marker to ensure nutritional ketosis is met (i.e., βHB > 0.5 mmol/L) [15]. βHB is easily measured via a finger prick and portable meter with single-use test strips. The measure only requires 1–2 drops of blood and reports βHB (mmol/L) within one minute. Acetoacetate (AcAc) is a ketone body of physiological importance, but it is more difficult to measure it than βHB due to its labile nature. Typically, AcAc is produced in the liver from acetyl-CoA from partial fatty acid oxidation [16] during starvation. AcAc and βHB interconvert through the ubiquitous beta-hydroxybutyrate dehydrogenase enzyme using NADH+H+ as a cofactor. AcAc serves as an intermediate form of ketones, which can spontaneously be converted to acetone or βHB for tissue delivery. Once in the target tissue, βHB is then converted to AcAc to generate mitochondrial acetyl-CoA to enter the citric acid cycle for ATP production [16]. If AcAc is unused by the cell due to a high NADH/NAD+ ratio, it could be converted back to βHB and/or be transported out, appearing in blood. Measuring circulating AcAc after exogenous βHB administration may be a marker of βHB usage by the peripheral tissues for energy.

AcAc is often measured via urine dipsticks as they are portable, affordable, and easy for the general population to use. Urinary measures of AcAc have shown poor correlation at higher ketone concentrations (>3.0 mmol/L) [17,18]. Accurately measuring circulating AcAc levels is important to assess what part of ketone bodies enters target tissues for ATP production and what part becomes acetone. To accurately measure AcAc concentration, sophisticated analytical methods can be used, specifically GC/MS (Gas Chromatography-Mass Spectrometry). As the sample is collected, AcAc is converted to βHB by reducing agents to preserve it from degradation. Given the complexity of analyses and availability of the GC/MS, circulating AcAc levels are not routinely measured and reported in this manner.

The few manuscripts where blood AcAc levels are reported used ketone esters, which have notable advantages in terms of efficacy and limiting gastrointestinal distress, but which may be prohibitively expensive for many people [19,20]. Ketone salts, which contain βHB, are a more feasible and cost-effective alternative to ketone esters, yet the ability of these supplements to induce increases in AcAc has not been formally addressed. Exogenous ketone supplements may be used to increase circulating ketone levels and consist of βHB [21]. Most studies of exogenous ketone supplements do not report on changes in AcAc levels following βHB consumption, making it unclear whether this conversion from βHB to AcAc occurs and to what extent AcAc increases. As mentioned, assessing AcAc levels is important to understand the balance and metabolism of ketones from exogenous sources. Furthermore, articles that have reported AcAc outcomes have used supplements that might not be feasible for regular use due to their cost and unpleasant taste [22]. As mentioned above, circulating AcAc levels in fed individuals given βHB supplements may serve as a marker of supplement utilization for energy. The purpose of our study was to determine if βHB salt supplementation will increase circulating AcAc levels in healthy individuals that are not consuming a ketogenic diet. We postulate that βHB salt supplementation will increase circulating AcAc levels as the peripheral tissues will only partially use it due to low ATP demand/TCA flux. A secondary purpose was to determine if any changes in AcAc were associated with changes in circulating βHB.

## 2. Materials and Methods

### 2.1. Experimental Design

A randomized, triple-blinded, placebo-controlled, cross-over design was used to determine circulating AcAc levels 30 min after consuming an exogenous racemic ketone salt (KS). The study consisted of one familiarization visit and two laboratory visits in which they consumed one of two supplements in a randomized order. A blinded statistician set the randomization order prior to the start of the study. The supplements included a racemic KS and a placebo; both were in powder form in a white packet labeled ‘A’ or ‘B’. The investigators and participants were blinded until the completion of the study. The KS included 7 g of racemic sodium D, L-βHB (50% D-βHB and 50% L-βHB) and a flavoring mixture. The placebo was flavor- and calorie-matched to the KS and included maltodextrin, sodium, and flavoring. The KS and placebo were not specific brands.

During the familiarization visit, participants read and signed an informed consent form, completed a health history questionnaire, and took a pregnancy test if female. They visited the laboratory two times after that for data collection, which were separated by a one-week washout period. The study was conducted in accordance with the Declaration of Helsinki and approved by the Institutional Review Board of Augusta University (IRBnet ID: 1317538, approved on 10 December 2018). Informed consent was obtained from all subjects involved in the study.

### 2.2. Participants

Participants included 16 young adults between the ages of 18–35 years old. Exclusion criteria included taking medications that affect blood pressure, insulin, or renal function, having metabolic syndrome factors such as type 2 diabetes, being pregnant, and/or indicating a pre-existing health condition on the health history questionnaire. Of the 16 participants, 13 were included in the statistical analysis. Participant characteristics are provided in Table 1.

### 2.3. Protocol

**Figure 1:**
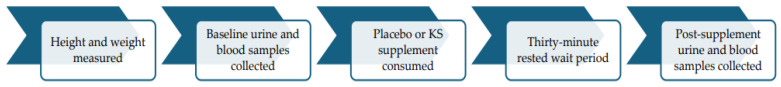
Both laboratory visits followed the same protocol with a one-week washout between visits.

Prior to both data collection visits, participants were asked to refrain from exercise, caffeine, and nicotine for 12 h and fast for 10 h. In addition, females took a pregnancy test prior to each data collection session. All measures were the same for both data collection visits. First, height and body mass were recorded. A urine sample was then collected to determine baseline urinary AcAc levels. After the urine sample, 250 µL of blood was collected via finger pricks and then processed for subsequent baseline plasma AcAc analysis. After baseline measures, participants drank either the placebo or KS supplement, which consisted of the powder mixed in 16 ounces of water. Thirty minutes after consuming the drink, the same urine and blood AcAc measures were taken. Figure 1 displays the process that occurred in both laboratory visits.

#### 2.3.1. Urinary AcAc Measure

Immediately after collecting each urine sample in a clean container, urinary AcAc was measured with a firm plastic strip with separate reagent areas (Siemens Urine Multistix 10 SG, Siemens Healthineers, Cary, NC, USA). The entire strip was immersed into the urine for 40 s and then removed carefully while running the edge of the strip against the rim of the container to remove excess urine. The strip was then held horizontally and lined up with the ketone color chart on the side of the bottle. The referenced ketone measured was specifically AcAc. The ketone color chart ranged from 5 to 160 mg/dL; specifically, 5 mg/dL indicated a trace amount, 15 mg/dL indicated a small amount, 40 mg/dL indicated a moderate amount, and 80–160 mg/dL indicated a large amount of AcAc. The AcAc value was visually interpreted according to the manufacturer’s instructions.

#### 2.3.2. Plasma AcAc and βHB Measurements

The 250 µL baseline and post-drink samples were immediately centrifuged for 10 min at 13,000 rpm at 4 °C. Plasma was then transferred into a tube containing NaB2H4 to convert plasma AcAc to deuterated beta-hydroxybutyrate ([2-2H]βHB). This step prevents degradation of AcAc. The processed samples were immediately frozen on dry ice and stored at −80 °C until analysis. Briefly, AcAc concentration was determined by determining the amount of generated [2-2H]βHB. Samples were thawed on ice and acidified by the addition of 1 N HCl. Care was taken to add acid slowly to prevent loss of deuterium from ΒHB. Internal standard of [2,4-13C2]βHB was added to samples and βHB was extracted by adding acetonitrile/Isopropanol mix (1:1, vol/vol) twice. Combined extracts were evaporated to dryness and βHB was converted to its trimethylsilyl (TMS) derivative by reacting lyophilized sample with 80 µL of bis (trimethylsilyl) trifluoroacetamide + 10% trimethylchlorosilane (Regis, Morton Grove, IL, USA) for 30 min at 75 °C. di-TMS derivative of βHB was analyzed by gas chromatography/mass spectrometry (GC/MS) using an Agilent 5973 mass spectrometer linked to a 6890 gas chromatograph equipped with an autosampler (Agilent Technologies, Santa Clara, California, USA). βHB (*m*/*z* 233) was detected under electron ionization mode. M1 ion (*m*/*z* 234) corresponding to [2-2H]βHB represented AcAc amount present in the sample after appropriate background subtraction. M2 ion (235) corresponding to an internal standard was monitored for quantification of AcAc using standard curve correction.

### 2.4. Data Analysis

All statistical analyses were performed using SPSS version 29 (IBM, Armonk, NY, USA). A predetermined alpha level of 0.05 was used for all analyses except where otherwise noted. Data were screened for outliers operationally defined as data points with a standardized value >3.0 from the group mean. Normality of each set of scores was assessed using the Shapiro–Wilk test, violations of which are reported in the Section 3. The assumption of sphericity was automatically met due to the inclusion of only two levels for each repeated-measures factor.

The combined effects of drink (Control and KS) and time (PRE and POST), on blood AcAc level were analyzed using a 2 × 2 repeated-measures ANOVA. Urinary ketone test results were analyzed in the same manner. Bonferroni-adjusted post hoc tests were performed as necessary to interpret significant interaction effects. Effect sizes for ANOVA results are reported as partial eta squared (η^2^) and interpreted according to the following benchmark values: small (η^2^ = 0.01–0.04), medium (η^2^ = 0.06–0.11), and large (η^2^ > 0.14) [23]. Effect sizes for post hoc tests are reported as Cohen’s d and interpreted as follows: small (d = 0.2–0.5), medium (d = 0.5–0.8), and large (d > 0.8) [23].

To supplement the ANOVA results for AcAc differences, a paired-sample *t*-test was conducted to compare the gain score (change in AcAc from PRE to POST timepoints) between the Control and KS conditions. The gain score analysis was performed to determine the effect size of the difference in AcAc change between conditions, which was reported as Cohen’s d and interpreted as previously described.

A bivariate Pearson correlation analysis was completed between the change in AcAc and the change in βHB from PRE to POST to assess the association between changes in plasma AcAc and βHB. Pearson’s r is reported to interpret the relationship as direct or indirect (positive or negative) and the strength of the relationship as small (0.1 ≤ r < 0.3), medium (0.3 ≤ r < 0.05), and large (0.05 ≤ r) [23].

The KS condition identified one outlier for the AcAc analysis at the PRE time point. Additionally, two subjects had at least one missing AcAc data point. All data for these three subjects was removed from all analyses. There was one subject with outlier values for urinary ketone readings in the Control condition at PRE and POST. All urinary ketone data for this subject was removed from this analysis only.

## 3. Results

For the AcAc data, the assumption of normality was violated for the Control condition at the PRE timepoint (*p* = 0.033) and the KS condition at the PRE timepoint (*p* = 0.013). For the urinary ketone analysis, the assumption of normality was violated in the KS group at the POST timepoint (*p* = 0.014). The ANOVA is robust to violations of normality; therefore, no adjustments were made to correct any reported violations of this assumption for ANOVAs [24].

**Figure 2:**
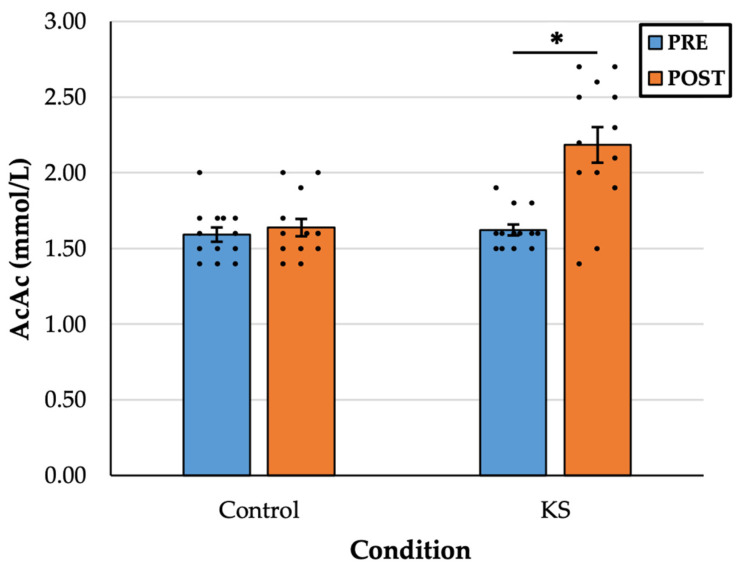
Blood concentration acetoacetate (AcAc) values before (PRE) and 30 min after (POST) the consumption of a control drink and a ketone salt (KS) supplement. Colored bars indicate sample means, whiskers indicate the corresponding standard error of the mean, and dots indicate individual data points. * = significant at the *p* < 0.05 level.

There was a statistically significant interaction between drink and time, F(1, 12) = 9.646, *p* = 0.009, η^2^ = 0.45. In the KS condition, there was a significant increase in AcAc from PRE (1.61 ± 0.12 mM) to POST (2.18 ± 0.41 mM), *p* < 0.001, d = 1.28. There was no difference in AcAc in the Control condition from PRE (1.59 ± 0.17 mM) to POST (1.66 ± 0.20 mM), *p* = 0.307, d = 0.30 (Figure 2).

**Figure 3:**
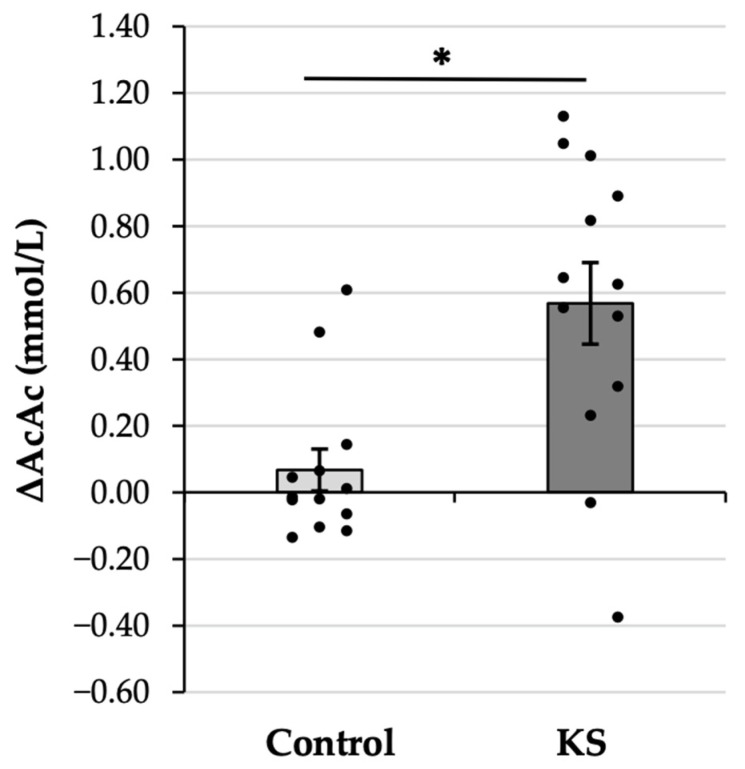
Gain scores representing the change in blood concentration of acetoacetate (ΔAcAc) values from before (PRE) to 30 min after (POST) the consumption of a control drink and a ketone salt (KS) supplement. Bars indicate sample means, whiskers indicate the corresponding standard error of the mean, and dots indicate individual data points. * = significant at the *p* < 0.05 level.

The AcAc gain score analysis was significant (Figure 3), with the 8X increase in AcAc in the KS condition as compared with the Control condition (KS: 0.57 ± 0.44 mM vs. Control: 0.07 ± 0.23 mM, *p* = 0.009). The observed effect was large (d = 0.86), verifying that the increase in AcAc following ketone ingestion was significantly and substantially larger than the increase that was observed following ingestion of a Control drink.

Expectedly, no urinary ketones were detected in the Control condition at any time point or in the KS condition at the PRE time point. There was a significant interaction effect, F(1, 11) = 10.210, *p* = 0.009, η^2^ = 0.48. Urinary ketones increased in the KS condition between PRE (0.00 ± 0.00 mg/dL) and POST (27.91 ± 29.05 mg/dL), *p* = 0.009, d = 0.92.

**Figure 4:**
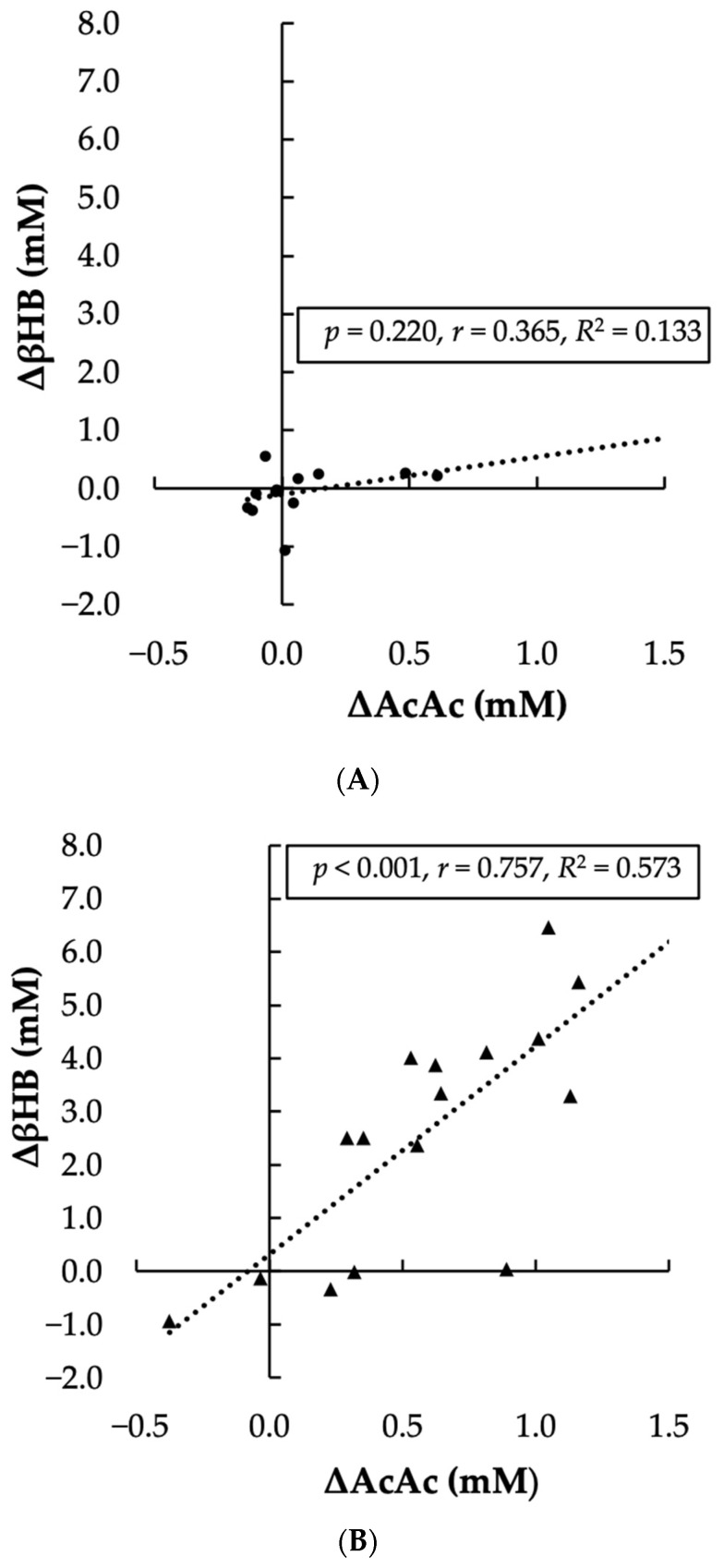
Scatterplot of blood concentration change in β-hydroxybutyrate (ΔβHB; y-axis) plotted against blood concentration change in acetoacetate (ΔAcAc; x-axis) from PRE to POST timepoints for (**A**) Control condition and (**B**) Ketone salt (KS) condition. Trendline and *p*, r, and R^2^ values are presented for condition.

The AcAc gain score in the Control condition violated the assumption of normality (*p* = 0.003). Given that Pearson’s correlation is robust to normality violations and that non-parametric alternative tests yielded the same outcome, we report the results of the Pearson correlation analysis here [25]. The linear relationship between the change in AcAc and the change in βHB was significant, positive, and large for the KS condition (*p* < 0.001, r = 0.757), but was non-significant, positive, and moderate for the Control condition (*p* = 0.220, r = 0.365). Visual representation of the association between AcAc and βHB in each condition is presented in Figure 4.

## 4. Discussion

This study aimed to determine if AcAc is increased following exogenous βHB salt supplementation. A secondary purpose was to determine if any changes in AcAc were associated with changes in βHB. As we postulated, consuming an exogenous βHB salt supplement resulted in a significant and marked increase in plasma AcAc. This suggests that βHB salt supplementation was only partially used for energy, and the rest was released as AcAc. The increase that was observed following βHB salt supplementation was significantly greater than that observed following consumption of placebo and was significantly and strongly related to the change in βHB. Together, these findings suggest that exogenous βHB from a racemic ketone salt supplement can be readily interconverted to AcAc, just as with ketone ester supplements.

βHB levels are an important indicator of nutritional ketosis, but the conversion of βHB to AcAc must occur to provide a bioenergetic benefit. Therefore, an accurate assessment of AcAc is imperative to fully evaluate ketone supplements’ efficacy. This is the first study to report on the changes in AcAc following racemic ketone salt ingestion using the GC/MS assessment method. This method is particularly advantageous because it preserves low amounts of plasma AcAc, otherwise subject to rapid oxidation. We exploited the fact that AcAc’s keto group can be converted to a hydroxyl group, namely βHB. We used isotopically labeled reducing reagent NaB2H4 to produce the conversion and introduce deuterium into AcAc to distinguish it from endogenous βHB in the sample. This strategy allowed us to quantify endogenous AcAc as well as βHB accurately.

Many studies reporting on the change in AcAc following exogenous ketone supplementation have used ketone esters. Leckey et al. (2017) observed a significant increase in AcAc following consumption of a ketone ester composed of a mixture of mono- and diesters in internationally competitive cyclists [19]. After drinking two doses of the supplement, participants completed an extensive warm-up and time trial of 31.17 km. AcAc remained higher than baseline from the time of supplement consumption until 60 min after the time trial was finished. McCarthy et al. (2021) similarly found that AcAc increased following the ingestion of a commercially available ketone monoester 30 min prior to a submaximal exercise bout [20]. AcAc was higher than when a placebo was ingested, and also increased throughout the cycling exercise period. Stubbs et al. (2017) assessed AcAc following consumption of a ketone monoester but only reported the comparison between AcAc in a fed state and a fasted state, noting that AcAc was not different [26]. The findings here build upon these earlier reports by confirming that AcAc is increased following supplementation with racemic ketone salts, which are more cost-effective and more commonly available than ketone esters. These supplements can therefore be considered more feasible for continued use than many ketone ester supplements. In addition, both exogenous ketone salts and esters may cause infrequent mild gastrointestinal effects, however, our participants did not complain of gastrointestinal discomfort after consuming either supplement (placebo or ketone salts) [27].

Cuenoud et al. (2020) assessed the change in AcAc following racemic ketone salt ingestion, in addition to other similar ketone supplements, and report findings similar to those observed here [28]. However, they did not measure AcAc directly. Total ketones and βHB were assessed via assay, and the difference between these values was deemed the AcAc value. Although theoretically sound, this method of quantifying AcAc may be subject to increased error since it relies on accurate measurement of two values (total ketones and βHB). Our findings corroborate those of Cuenoud et al. (2020) that AcAc is increased following ketone salt supplementation but may be considered a more accurate and complete record of this change. One notable discrepancy between findings is that compared to results reported by Cuenoud et al. (2020), participants in the current study achieved substantially higher peak AcAc values (2.18 mM vs. 0.23 mM) and increases in AcAc (0.57 mM vs. 0.18 mM, even though the dose of βHB pro-vided here was roughly half of that provided to participants by Cuenoud et al. (2020). The discrepancy in AcAc values across time points could have been due to differences in protocol, as Ceunoud et al. provided a standard meal to participants while those in the current study were fasted for 10 h. However, it has been reported that plasma AcAc changes following a standardized dose of exogenous ketones are not different between a fed and fasted state [26]. This discrepancy was likely caused by measurement methodologies, with assay or meter devices yielding results that underestimate the true outcome of KS supplementation on AcAc.

**Figure 5:**
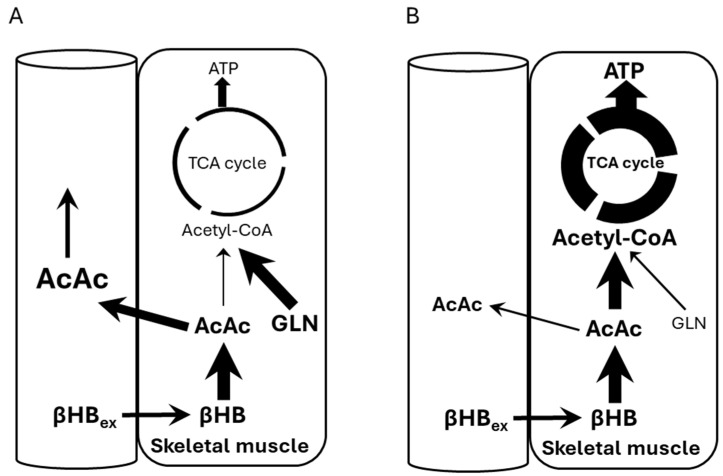
Metabolism of exogenous βHB. Font size and arrow thickness represents relative carbon flux levels. Panel (**A**): At rest, low ATP turnover/Krebs cycle flux increases plasma AcAc generation. Panel (**B**): During exercise, high ATP turnover/Krebs cycle flux will use βHB towards energy, thus lowering plasma AcAc.

Figure 5 shows potential mechanisms explaining our observations. Panel A shows our subjects who were at rest and their ATP demand/Krebs cycle flux was low, while Panel B shows a scenario of subjects under high ATP demand, i.e., exercise. Under rest conditions, the NADH/NAD+ ratio is high, thus preventing BHB conversion to AcAc and allowing glycogen usage. This shift causes some AcAc to be transported out, raising its circulating levels. In Panel B, ATP demand increases (exercise) thus decreasing the NADH/NAD+ ratio and pulling βHB toward AcAc and into Krebs cycle.

Regarding the secondary purpose of the study, we found that the change in βHB and change in AcAc levels from baseline to post-supplementation showed a strong positive association. βHB must be converted to AcAc before metabolizing it in the muscle. In order for the consumed KS supplement to yield some bioenergetic benefit, this conversion must take place. Although these data and their analysis do not support a causal relationship between βHB consumption and ensuing conversion to AcAc, the strong positive relationship observed supports this mechanism of increased AcAc availability as a result of exogenous βHB supplementation.

Importantly, participants in the current study were untrained healthy individuals who were not adapted to the ketogenic diet. As such, they most likely did not possess the increased levels of ketolytic enzymes (i.e., beta-hydroxybutyrate dehydrogenase) that are common following endurance training (for review see Evans et al., 2017) in athletic populations reported on previously [19,20,26,29]. These findings corroborate those of Cuenoud et al. (2020), together suggesting that any bioenergetic benefit of consuming exogenous βHB is not restricted to highly trained competitive athletes [28]. Furthermore, this demonstrates that racemic ketone salts may potentially elevate circulating ketone levels to impact overall health and function in untrained individuals on a normal diet. In addition to physical performance, elevated ketones are associated with improved cognitive performance and associated cognitive impairments such as Alzheimer’s Disease [13,14]. Improvements in metabolic health such as improved glucose tolerance and insulin sensitivity and reduced blood pressure, circulating triglycerides, and inflammation may also occur with elevated ketone levels, thereby, improving related conditions such as cardiovascular disease and type 2 diabetes [10,11,12].

The findings from this study are practically meaningful because they confirm that the benefits of ketone ester supplementation listed above can be achieved, to a certain degree, with ketone salt use. Ketone salts are generally more palatable than ketone esters and are substantially cheaper. People seeking the documented benefits of ketone supplementation, without adherence to a restrictive ketogenic diet, can do so for a fraction of the cost of ketone ester supplements.

Substantial individual variations in AcAc values were observed, as not all participants increased AcAc following ingestion of ketone salts. One person experienced no increase in AcAc, and one person experienced a decrease in AcAc. Variations in ketolytic enzymes could be responsible for this lack of uniform response. Other demographic factors may have influenced the changes in ketones that were observed, such as obesity level and muscle mass [30]. To address this possibility, post hoc correlational analyses were performed between the changes in ketones (AcAc and βHB) and the variables BMI, total body mass, and age. Partial correlation coefficient analyses were also performed to control for the potential effect of biological sex. In all cases, correlation coefficients ranged from small to medium and were insignificant as presented in Supplementary Tables S1 and S2. The influence of these variables on ketone body kinetics cannot be ruled out given the small sample size, but this issue of individual variation merits attention from future research works to ensure effective dosing guidelines.

One limitation of this study that was previously mentioned was that we cannot verify whether the increase in AcAc that was observed was due to the βHB that was ingested. Tracer techniques, such as that used by Cuenoud et al. (2020) have been used in the past to observe the metabolism of ketones [28] more clearly. Alternatively, isotopically labeled KS could have been used. However, the cost of such a mixture is prohibitive. Use of a correlation analysis to show that the changes in βHB (from baseline) and changes in AcAc from baseline were significantly and strongly related lends indirect evidence that the supplement consumption caused the observed outcome. Another limitation was that the supplement dose provided to participants was not standardized to body mass or muscle mass. This could lead to some of the individual variation in AcAc production noted earlier. We provided an absolute dose equivalent to one serving (packet) of the supplement because this is the likely dosing behavior that people engage in in practice. Another potential limitation of the findings of this study is the relatively small sample size. Although power levels >0.80 were observed for all primary findings reported, larger-scale studies with more participants are needed to fully investigate the effects of different ketone supplements on bioenergetic variables of interest.

The main strength of the study was the accurate quantification of endogenous AcAc by first preserving it by chemically converting to deuterated βHB and then determining its concentration distinguishing it from endogenous βHB. GC/MS is considered a leading methodology for determining circulating metabolites like ketones. It is a high throughput, robust, and accurate way to precisely quantify small molecules, i.e., ketones because GC/MS is able to precisely identify molecules from complex biological matrix-like plasma based on their retention time and fragmentation pattern, unique for each molecule. GC/MS sensitivity has improved drastically since its introduction into analytical chemistry, biochemistry, and biomedicine. Lastly, due to its robustness, GC/MS popularity and availability have improved while cost of analysis steadily decreased.

Other strengths relate to the specific study design. Using a repeated-measures randomized crossover design ensured that extraneous error from individual responses was limited. Double-blind administration of the KS and placebo supplements lend further strength to the study results by limiting bias on the part of participants and investigators.

Future studies can incorporate the findings we present here by assessing AcAc with the most accurate measurement techniques available along with βHB, given that both values are relevant to evaluating the efficacy of ketone supplements and the ketogenic diet in general.

## 5. Conclusions

Consuming an exogenous βHB salt supplement resulted in increased AcAc levels, as measured with GC/MS, which was strongly related to the increased βHB levels. Exogenous βHB from a racemic ketone salt supplement can be converted to AcAc within 30 min, just as with ketone ester supplements.

## Figures and Tables

**Table 1:** Participant characteristics (mean ± standard deviation) from 13 participants included in the analysis.

Characteristic	Men (n = 7)	Women (n = 6)	Total (N = 13)
Age (years)	22.6 ± 5.7	20.5 ± 1.5	21.6 ± 4.3
Height (cm)	186.5 ± 11.6	168.4 ± 6.9	178.2 ± 13.2
Weight (kg)	83.7 ± 13.7	77.1 ± 21.4	80.6 ± 17.2
BMI (kg/m^2^)	24.4 ± 5.9	27.2 ± 7.2	25.7 ± 6.4

Note: BMI = body mass index.

## Data Availability

Complete descriptive data and statistical results can be found at an online repository using the following link, which was last updated on 6 May 2025: https://osf.io/gd7z4/.

## Supplementary Materials

The following supporting information can be downloaded at: https://www.mdpi.com/article/10.3390/nu17101665/s1, Supplementary Table S1. Zero-order correlation analysis results for the relationships between demographic variables and the changes in ketones (from pre to post time points) in the ketone salt condition.; Supplementary Table S2. Partial correlation analysis results (controlling for the effect of biological sex: male or female) for the relationships between demographic variables and the changes in ketones (from pre to post time points) in the ketone salt condition.
