# Supplementary material for: Effectiveness of Exogenous Ketone Salts in Enhancing Circulating Acetoacetate Levels—A Pilot Study in Healthy Adults
Source: Nutrients. 2025 May 14;17(10):1665. doi: 10.3390/nu17101665 (PMC12113729; doi:10.3390/nu17101665)
Supplement: Supplementary file 1 [file nutrients-17-01665-s001.zip › nutrients-3562415-supplementary.pdf]

Supplementary Table S1. Zero-order correlation analysis results for the relationships between demographic variables and the changes in ketones (from pre to post time points) in the ketone salt condition. BMI = body mass index; ΔAcAc = the change in circulating acetoacetate; ΔBHB = the change in circulating beta-hydroxybutyrate.

	Body mass (kg)		BMI (kg/m^2)		Age (years)	
	r	p	r	p	r	p
ΔAcAc	0.292	0.333	-0.073	0.813	0.052	0.865
ΔBHB	-0.081	0.792	-0.410	0.164	0.169	0.582

Supplementary Table S2. Partial correlation analysis results (controlling for the effect of biological sex: male or female) for the relationships between demographic variables and the changes in ketones (from pre to post time points) in the ketone salt condition. BMI = body mass index; ΔAcAc = the change in circulating acetoacetate; ΔBHB = the change in circulating beta-hydroxybutyrate.

	Body mass (kg)		BMI (kg/m^2)		Age (years)	
	$r_{partial}$	$p_{partial}$	$r_{partial}$	$p_{partial}$	$r_{partial}$	$p_{partial}$
ΔAcAc	0.189	0.555	0.004	0.991	-0.074	0.819
ΔBHB	-0.283	0.373	-0.378	0.225	0.038	0.906
